# Effects of 2-Methoxyestradiol, a Main Metabolite of Estradiol on Hepatic ABCA1 Expression in HepG2 Cells

**DOI:** 10.3390/nu14020288

**Published:** 2022-01-11

**Authors:** Tomohiro Ibata, Jingya Lyu, Hitomi Imachi, Kensaku Fukunaga, Seisuke Sato, Toshihiro Kobayashi, Takanobu Saheki, Takafumi Yoshimura, Koji Murao

**Affiliations:** 1 Department of Endocrinology and Metabolism, Faculty of Medicine, Kagawa University, 1750-1 Ikenobe, Miki-cho 761-0793, Kagawa, Japan; ibata.tomohiro@kagawa-u.ac.jp (T.I.); ljy2020@jnu.edu.cn (J.L.); imachi.hitomi@kagawa-u.ac.jp (H.I.); Fukunaga.kensaku@kagawa-u.ac.jp (K.F.); sato.seisuke@kagawa-u.ac.jp (S.S.); kobayashi.toshihiro@kagawa-u.ac.jp (T.K.); saheki.takanobu@kagawa-u.ac.jp (T.S.); yoshimura.takafumi@kagawa-u.ac.jp (T.Y.); 2 Department of Physiology, School of Medicine, Jinan University, 601 Huangpu Avenue West, Tianhe District, Guangzhou 510632, China

**Keywords:** ABCA1, 2-methoxyestradiol, lipid accumulation, fatty liver, cell signaling

## Abstract

ATP-binding cassette transporter A1 (ABCA1) is a key regulator of lipid efflux, and the absence of ABCA1 induces hepatic lipid accumulation, which is one of the major causes of fatty liver. 2-Methoxyestradiol (2-ME_2_) has been demonstrated to protect against fatty liver. In this study, we investigated the effects of 2-ME_2_ on the hepatic lipid content and ABCA1 expression. We found that 2-ME_2_ dose-dependently increased ABCA1 expression, and therefore, the lipid content was significantly decreased in HepG2 cells. 2-ME_2_ enhanced the ABCA1 promoter activity; however, this effect was reduced after the inhibition of the PI3K pathway. The overexpression of Akt or p110 induced ABCA1 promoter activity, while dominant-negative Akt diminished the ability of 2-ME_2_ on ABCA1 promoter activity. Further, 2-ME_2_ stimulated the rapid phosphorylation of Akt and FoxO1 and reduced the nuclear accumulation of FoxO1. Chromatin immunoprecipitation confirmed that FoxO1 bonded to the ABCA1 promoter region. The binding was reduced by 2-ME_2_, which facilitated ABCA1 gene transcription. Furthermore, mutating FoxO1-binding sites in the ABCA1 promoter region or treatment with FoxO1-specific siRNA disrupted the effect of 2-ME_2_ on ABCA1 expression. All of our results demonstrated that 2-ME_2_ might upregulate ABCA1 expression via the PI3K/Akt/FoxO1 pathway, which thus reduces the lipid content in hepatocytes.

## 1. Introduction

2-Methoxyestradiol (2-ME_2_) is a main metabolite of estradiol and is generated by a sequential process: 17β-estradiol (E2) produces 2-hydroxyestradiol (2HE) via the enzyme cytochrome P450 isoform 1A1, and then, 2HE generates 2-ME_2_ by a conjugation reaction catalyzed via the enzyme catechol-*O*-methyltransferase (COMT) [1]. Estrogen protects women from NAFLD before menopause by reducing the TG accumulation in the liver via the activation of the estrogen receptor (ER), which is the main receptor of E2, or the G protein-coupled estrogen receptor (GPER or Gpr30), a receptor of 2-ME_2_ [2]. Differently from E2, 2-ME_2_ has a very low ability of binding to classical ER, indicating a lack of cytotoxicity [3,4].

As an important lipid transporter, ATP-binding cassette transporter A1 (ABCA1) is a 254-kD membrane protein to transport cholesterol from the cytoplasm to apolipoproteins, and it plays a critical role in the process of reverse cholesterol transport [5]. ABCA1 is firstly detected as a mutated molecule in patients with Tangier disease, and the reduction of ABCA1 results in a severe deficiency of high-density lipoprotein (HDL) and cholesterol accumulation in the liver [6]. Several reports have indicated that the secretion of TG and accumulation of lipids in hepatocytes is significantly increased after blocking the ABCA1 function, pointing out the close relationship between ABCA1 expression and fatty liver disease [7,8].

The liver is an important organ that performs varying metabolic functions, including the metabolism of lipids. As part of multiple lipid-processing functions performed in hepatocytes, overproduced lipids in the cytoplasm are normally stored in triglyceride (TG)-enriched lipoproteins, such as low-density lipoprotein cholesterol (LDL-C) and very low-density lipoprotein cholesterol (VLDL-C) in the cytoplasm. Abnormal lipid accumulation in hepatocytes leads to several metabolic diseases, including non-alcoholic fatty liver disease (NAFLD) [9]. Recently, 2-ME_2_ has been proven to protect the liver from ischemia/reperfusion-induced damage in alcoholic fatty livers [10]. However, little is known about the role of 2-ME_2_ in regulating the hepatic lipid content. In the present study, we studied the detailed mechanisms of 2-ME_2_-stimulating ABCA1 expression in HepG2 cells.

## 2. Materials and Methods

### 2.1. Cell Culture

HepG2 cells (Riken Cell Bank, Japan) were cultured in Dulbecco’s modified Eagle’s minimal essential medium with glucose of 1000 mg/L (L-DMEM; Gibco) supplemented with 10% heat-inactivated certified fetal bovine serum (Thermo Scientific; Scoresby VIC, Australia), 0.1-mg/mL streptomycin, and 100-U/mL penicillin, as described previously [11]. For treatment, HepG2 cells were starved in phenol-free L-DMEM supplemented with 0.5% charcoal/dextran-treated FBS for 6 h and then were treated with 2-ME_2_ at varying doses (0.01, 0.1, 1, and 10 μM) for 24 h or 2-ME2 at 1 μM for varying times (5, 10,15, and 30 min). For treatment with inhibitors, after 6 h of starvation, HepG2 cells were firstly treated with PI3K inhibitor LY294002 at 10 μM, MEK/ERK inhibitor PD98059 at 10 μM, or PKC inhibitor bisindolylmaleimide I at 1 μM for 30 min and then were incubated with 2-ME2 at 1 μM for 24 h.

### 2.2. Western Blot Analysis

The total proteins and nuclear proteins were extracted from HepG2 cells, as described previously [12]. Concentrations of the proteins were determined with GeneQuant 1300.

As described previously, after separation by 7.5% sodium dodecyl sulfate (SDS)-polyacrylamide gel, the proteins were transferred to polyvinylidene difluoride membranes for immunoblotting [13]. The membranes with proteins were blocked with skim milk overnight and then incubated with 3% BSA diluted in PBS with 0.1% Tween 20 (PBS-T) containing rabbit polyclonal antibody, the first antibody against ABCA1 (1:500; Santa Cruz, sc-20794), Akt (Upstate, 28740; 1:1000), FoxO1 (1:1000; CST, 2880s), p-Akt Ser 473 (CST, 9271s; 1:1000), p-FoxO1 Ser256 (1:1000; CST, 9461s), GAPDH (1:5000; TREVIGEN, 2275-PC-100), or nuclear TFIID (1:200; Santa Cruz, sc-273) overnight and incubated with horseradish peroxidase (HRP)-linked antirabbit IgG secondary antibody (1:2000; DakoCytomation, P0448) for 1 h at 4 °C. Then, the targeted proteins were detected by ECL (GE Healthcare). The analysis of the protein bands by Western blot was carried out under a Luminescent image analyzer LAS-1000 Plus (Fuji Film, Japan).

### 2.3. Reverse Transcription-Quantitative Real-Time Reverse Transcriptase-Polymerase Chain Reaction (RT-qPCR)

Total RNA was extracted from HepG2 cells treated with 2-ME_2_ at varying doses for 24 h in RNA-Bee reagent, according to the manufacturer’s protocols. Each sample was repeated 3 times. We synthesized cDNA from 1 μg of total RNA with SuperScript II reverse transcriptase (Invitrogen), according to the manufacturer’s protocols. PCR was performed with a final volume of a 10-µL mixture of cDNA and LightCycler 480 SYBR Green Master (Roche) by using CFX96 Touch Real-Time PCR Detection Systems (Bio-Rad), as described previously [14]. The sense and antisense primers were shown as the following: human ABCA1 5′-AACAGTTTGTGGCCCTTTTG-3′ and 5′-AGTTCCAGGCTGGGGTACTT-3′ [15]; human GAPDH 5′-ATGGGGAAGGTGAAGGTCG-3′ and 5′-GGGGTCATTGATGGCAACAATA-3′. All primers used were amplified in the same PCR conditions using 60 °C as the annealing temperature. In each set of PCR reactions, we used water as the negative control and 5 dilutions of the control cDNA as the standards. Each sample was repeated 3 times with human GAPDH as the reference gene, as described previously [11].

### 2.4. Transfection and Luciferase Reporter Gene Assay

The human ABCA1 promoter region was constructed with luciferase reporter plasmid (pABCA1-LUC), and FoxO1 response sequence (FRS)-mutated plasmid (pABCA1-mt-LUC) was generated by a site-directed mutagenesis kit, as described previously [16,17]. Purified promoter plasmid was transfected into HepG2 cells using Lipofectamine (Life Technologies, Gaithersburg, MD, USA). For co-transfection, a vector expressing the p110 catalytic subunit of PI3K (p110), Akt, or the dominant-negative mutant of Akt (Akt with a K197M mutation, Akt-DN) was co-transfected with pABCA1-LUC using Lipofectamine in HepG2 cells. Cells transfected with pABCA1-LUC were treated with or without 2-ME_2_ at 1 µM for 24 h before harvest. HepG2 cells without plasmid-transfection were used as a normalization of each experiment. The luciferase activity was checked in an aliquot of the cytoplasmic preparation, as described in the manufacturer’s instructions (ToykoInk, Tokyo, Japan) by a microplate reader (SH-9000Lab; CORONA), as previously described [17]. One microgram of Rous sarcoma virus-β-galactosidase (RSV-β Gal) plasmid was added to all transfections to monitor the efficiency of the DNA uptake by HepG2 cells, as described in a previous study [18]. All assays were corrected for β-galactosidase activity, and the total amount of protein in each reaction was identical.

### 2.5. Cholesterol and Triglyceride Content Assay

To measure the total cholesterol and triglyceride concentration, the cells were washed by PBS and lysed by HEPES buffer with 1% triton X-100. A colorimetric assay was used to measure the cholesterol content, which was carried out by random-access chemistry analyzer ARCHITECT c8000 with reagents widely combined with cholesterol [17].

### 2.6. Oil Red O Staining

HepG2 cells were seeded on coverslips and then were treated with 2-ME2 for 24 h. Cells were fixed by 4% paraformaldehyde (PFA) for 30 min at room temperature and washed by PBS, pH 7.2 three times. Fixed cells were incubated with Oil Red O working solution for 15 min. After washing three times with PBS, the nucleus was stained with hematoxylin solution for 30 s. After washing three times with PBS again, the cells were mounted, and lipid droplets in the cells were monitored by an Olympus upright microscope (BX-51/DP-72).

### 2.7. Chromatin Immunoprecipitation Assay

The chromatin immunoprecipitation (ChIP) assay was performed by the ChIP-IT^TM^ kit (Active Motif). Chromatin was immunoprecipitated with 2 μg of antibody of either FoxO1, TFIID, or control IgG. The harvested DNA was detected by real-time PCR and PCR to harvest the FRS fragment from −641 to −472 in the human ABCA1 promoter sequence by using sense and antisense primers 5′-AATCTCCAAGGCAGTAGGTCG-3′ and 5′-GAATCTCCCTCAGGACGCCAA-3′ [17]. PCR was performed with the TAKARA PCR thermal cycler MP in the amplification program: 95 °C for 5 min, followed by 36 cycles of 95 °C for 20 s, 61 °C for 30 s, and 72 °C for 30 s. The products of the PCR (169 bp) were detected by 3% agarose gel electrophoresis.

### 2.8. Transfection of Small Interfering RNA (siRNA)

siRNAs were designed to target the following cDNA sequences: scrambled, 5′-GGCTTATTGTTCTTAGTAAGA-3′; FoxO1-siRNA, 5′-AATGGCGTGCACTTTCTGCAG-3′ [17]. The transfection of siRNA was performed by using siPORT Lipid (Ambion), as described in a previous study [16].

### 2.9. Statistical Analysis

Data were represented as the mean ± SEM (*n* = 3) of separate experiments for each group. Statistical analyses were performed using Excel software and SPSS software. Statistical comparisons were made by one-way ANOVA or Student’s *t*-test; *p* < 0.05 was considered significant.

## 3. Results

### 3.1. 2-Methoxyestradiol (2-ME_2_) Upregulates the Expression of ABCA1 in HepG2 Cells

**Figure 1:**
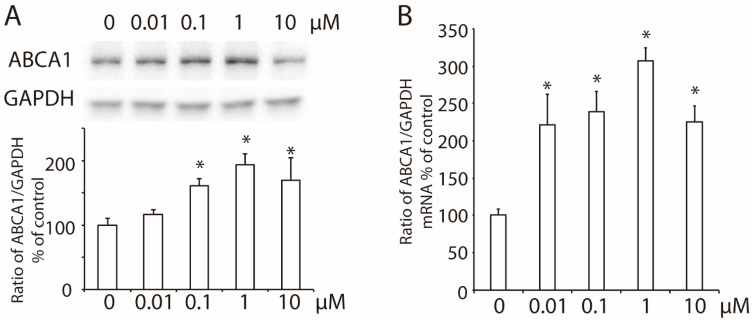
Effects of 2-ME_2_ on the expression of ABCA1 in HepG2 cells. (**A**) ABCA1 protein expression in HepG2 cells treated with 2-ME_2_ at varying doses for 24 h. (**B**) Effect of 2-ME_2_ on the mRNA level of ABCA1 expression. An abundance of GAPDH served as the control, and the relative expression of ABCA1 to GAPDH is shown as a percent of the control in the histogram. The histogram shows the mean ± SEM (*n* = 3) of separate experiments for each group. * *p* < 0.05 compared to 0.

To check the effect of 2-ME_2_ on hepatic ABCA1 expression, we treated HepG2 cells with 2-ME_2_ at varying concentrations (0–10 μM) for 24 h. The results showed that both the protein level and mRNA levels of hepatic ABCA1 increased with an increase in the dose of 2-ME_2_ (Figure 1A,B).

### 3.2. 2-ME_2_ Enhances the Promoter Activity of ABCA1 via the PI3K/Akt Pathway in HepG2 Cells

**Figure 2:**
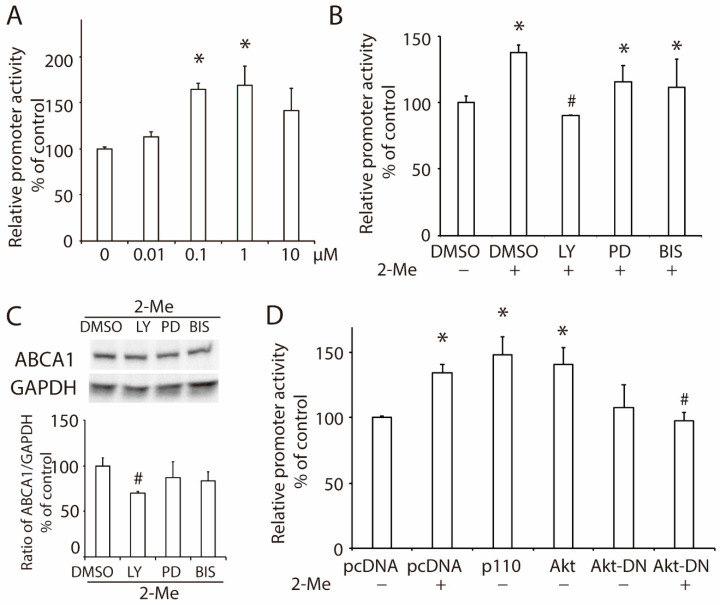
2-ME_2_ enhances the promoter activity of ABCA1 via the PI3K/Akt signaling pathway. (**A**) ABCA1 promoter activity in HepG2 cells treated with 2-ME_2_ at varying doses for 24 h. (**B**) Effects of a PI3K inhibitor LY294002 (LY), an MEK/ERK inhibitor PD98059 (PD), or a PKC inhibitor bisindolylmaleimide I (BIS) on 2-ME_2_-induced ABCA1 promoter activity in HepG2 cells. (**C**) Protein expression of ABCA1 in HepG2 cells treated with 2-ME_2_ with or without LY, PD, or BIS. (**D**) Role of the PI3K/Akt pathway on ABCA1 promoter activity induced by 2-ME_2_. pcDNA, control vector; p110, overexpression of the p110 catalytic subunit of PI3K; Akt, overexpression of Akt; Akt-DN, domain-negative Akt. Each data shows the mean ± SEM (*n* = 3) of separate experiments for each group. * *p* < 0.05 compared to control or pcDNA; # *p* < 0.05 compared to 2-ME_2_.

A luciferase reporter system constructed with an ABCA1 promoter (pABCA1-LUC) was employed to investigate the role of 2-ME_2_ in ABCA1 transcription. Similar to that in ABCA1 expression, 2-ME_2_ significantly enhanced ABCA1 promoter activity, and the strongest promoter activity was induced by 1 μM of 2-ME_2_ (Figure 2A). To detect the pathways in 2-ME_2_-mediated ABCA1 promoter activity, we treated HepG2 cells with inhibitors that separately block PI3K (10-μM LY294002), MEK/ERK (10-μM PD98059), or PKC (1-μM bisindolylmaleimide I) before the 2-ME_2_ treatment. As shown in Figure 2B, the inhibitors of MEK/ERK or PKC did not alter the action of 2-ME_2_, while LY, the inhibitor of PI3K, blocked the enhancement of ABCA1 promoter activity by 2-ME_2_. This result was confirmed by Western blotting that the inhibition of PI3K with LY blocked 2-ME_2_-upregulated ABCA1 expression, while the other two inhibitors did not significantly affect ABCA1 expression, suggesting that 2-ME_2_ stimulates ABCA1 promoter activity via the PI3K pathway. Next, we overexpressed the p110 catalytic subunit of PI3K or Akt, which are downstream of the PI3K cascade pathway, in HepG2 cells, and the ABCA1 promoter activity was enhanced significantly (Figure 2D). However, domain-negative Akt (Akt-DN) reduced the ABCA1 promoter activity enhanced by 2-ME_2_ (Figure 2D). These results demonstrated that 2-ME_2_-induced ABCA1 transcription required activation of the PI3K/Akt signaling pathway.

### 3.3. 2-ME_2_ Reduces Lipid Content via the PI3K Pathway in HepG2 Cells

**Figure 3:**
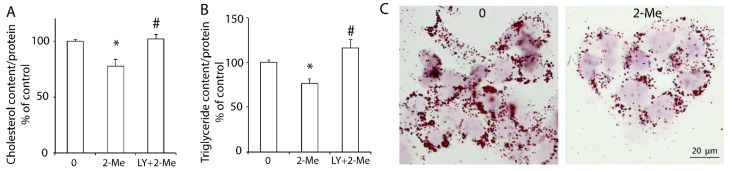
2-ME_2_ decreases the lipid content in HepG2 cells via the PI3K pathway. Cholesterol content (**A**), triglyceride content (**B**), or Oil Red O staining (**C**) in HepG2 cells with varying treatments. The cholesterol content was normalized by the protein concentration. 2-ME_2_, 2-ME_2_ at 1 μM; LY, PI3K inhibitor LY294002 at 10 μM. The results are represented as the mean ± SEM (*n* = 3) of separate experiments for each group. * *p* < 0.05 compared to control; # *p* < 0.05 compared to 2-ME_2_.

As a cholesterol exporter, ABCA1 exports intracellular cholesterol to HDL in the presence of ApoA-I. Our previous study showed that the upregulation of hepatic ABCA1 induced by insulin-like growth factor-1 (IGF-1) or glucagon-like peptide-1 (GLP-1) decreased the lipid content in HepG2 cells [19,20]. In this study, we demonstrated that 2-ME_2_ significantly reduced the cholesterol content to 78 ± 6% of that in the control group. However, blocking the PI3K pathway by its specific inhibitor, LY, canceled this reduction (Figure 3A). Further, the triglyceride content was significantly decreased by 2-ME_2_, which was blocked by LY (Figure 3B). Consistently, Oil Red O staining also showed that HepG2 cells with 2-ME_2_ treatment had fewer and smaller lipid droplets compared to the control group (Figure 3C). These data indicated that 2-ME_2_ could reduce the hepatic lipid content via the PI3K pathway.

### 3.4. 2-ME_2_ Stimulates the Activation of Akt and Transcription Factor FoxO1 in HepG2 Cells

**Figure 4:**
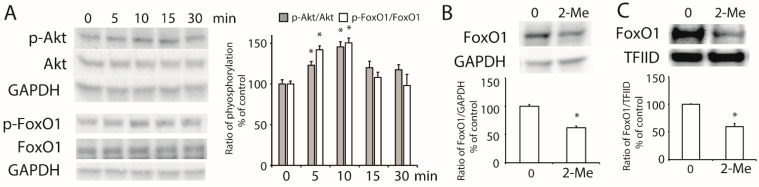
Activation of Akt and FoxO1 induced by 2-ME2. (**A**) 2-ME_2_ stimulated the phosphorylation of Akt at the Ser473 site and FoxO1 at the Ser256 site. A histogram shows the mean ± SEM (*n* = 3) of phosphorylation for each group. (**B**) Effect of 2-ME_2_ on the expression of FoxO1. (**C**) Nuclear abundance of FoxO1 in HepG2 cells treated with 2-ME_2_. Abundance of GAPDH or TFIID served as the control. A histogram shows the mean ± SEM (*n* = 3) of separate experiments for each group. * *p* < 0.05 compared to 0.

To further study the activation of Akt, HepG2 cells were treated with 1 μM of 2-ME_2_ for different time periods. As shown in Figure 4A, the phosphorylation of Akt was detected at 5 min and 15 min after the 2-ME_2_ treatment. Downstream of the PI3K/Akt pathway, FoxO1 belongs to the FoxO transcription factor family responding to the activation of Akt [21]. Next, we examined the role of FoxO1 in the effect of 2-ME_2_ on ABCA1 expression. The results showed that the phosphorylation of FoxO1 was stimulated by 2-ME_2_ from 5 min to 15 min (Figure 4A). Moreover, treatment with 2-ME_2_ for 24 h decreased the expression of FoxO1 in HepG2 cells (Figure 4B). Once FoxO1 was phosphorylated, it was translocated from the nucleus to the cytoplasm, which allowed stimulating ABCA1 gene transcription. As shown in Figure 4C, the nuclear abundance of FoxO1 was reduced by 2-ME_2_. These results demonstrated that 2-ME_2_ activated the phosphorylation of Akt and FoxO1, which may contribute to the regulation of ABCA1 transcription.

### 3.5. 2-ME_2_ Increases ABCA1 Transcription via FoxO1

**Figure 5:**
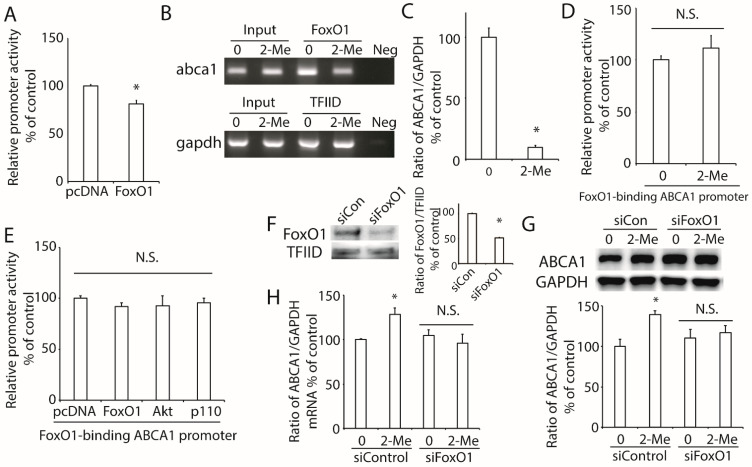
Role of FoxO1 in 2-ME_2_-mediated ABCA1 transcription in HepG2 cells. (**A**) Effect of FoxO1 on ABCA1 promoter activity. (**B**) Binding of FoxO1 to the ABCA1 promoter region. FoxO1 specifically immunoprecipitates ABCA1 chromatin by the ChIP assay. No ChIP was detected when the chromatin was immunoprecipitated with an unspecific negative control IgG (IgG). (**C**) ChIP-real time PCR showed that 2-ME_2_ reduced the binding of FoxO1 to ABCA1 promoter. (**D**), Effect of 2-ME_2_ on FoxO1-binding sites-mutated promoter activity (5′-AACA-3′ to 5′-GGAG-3′). (**E**) Effect of FoxO1, Akt, or p110 on FoxO1-binding sites-mutated promoter activity. (**F**) Expression of nuclear FoxO1 after silencing FoxO1 with specific siRNA. (**G**,**H**) Effect of 2-ME_2_ on the protein (**G**) and mRNA (**H**) expression of ABCA1 after silencing FoxO1. siCon, scramble siRNA; siFoxO1, FoxO1-specific siRNA. Each data point shows the mean ± SEM (*n* = 3) of separate experiments for each group. * *p* < 0.05 compared to 0; N.S., no significant difference.

To study the effect of FoxO1 on the transcription of ABCA1, we co-transfected the plasmid of FoxO1 and pABCA1-LUC into HepG2 cells. As shown in Figure 5A, the overexpression of FoxO1 reduced the ABCA1 promoter activity. Previously, we reported that there is a FoxO1 response sequence (FRS) motif in the ABCA1 promoter region [16]. Based on the results of the ChIP assay, we confirmed that FoxO1 directly bonded to the ABCA1 promoter region (Figure 5B), and ChIP real-time PCR demonstrated that this binding was significantly decreased by the treatment with 2-ME_2_ (Figure 5C). Next, we generated a luciferase-reporter plasmid with a mutation of the FRS motif in the ABCA1 promoter (FoxO1-binding-mutated ABCA1 promoter) by site-directed mutagenesis and found that 2-ME_2_ did not alter the FoxO1-mutated promoter activity (Figure 5D). Moreover, the overexpression of FoxO1, p110, or Akt could not affect the FoxO1-binding-mutated ABCA1 promoter activity (Figure 5E), suggesting that the FRS motif is required in 2-ME_2_-regulated ABCA1 transcription.

To further examine the effect of FoxO1 on the regulation of ABCA1 expression, we tried to silence FoxO1 expression by its specific siRNA in HepG2 cells. The results showed that the expression of FoxO1 was remarkably decreased by FoxO1-specific siRNA but not by a scrambled siRNA (Figure 5F). Then, we treated these HepG2 cells with 2-ME_2_ and found that the protein and mRNA levels of ABCA1 were not changed by 2-ME_2_ after the silencing of FoxO1 (Figure 5G,H).

These findings support the idea that FoxO1 is critical for 2-ME_2_-stimulated ABCA1 expression.

## 4. Discussion

**Figure 6:**
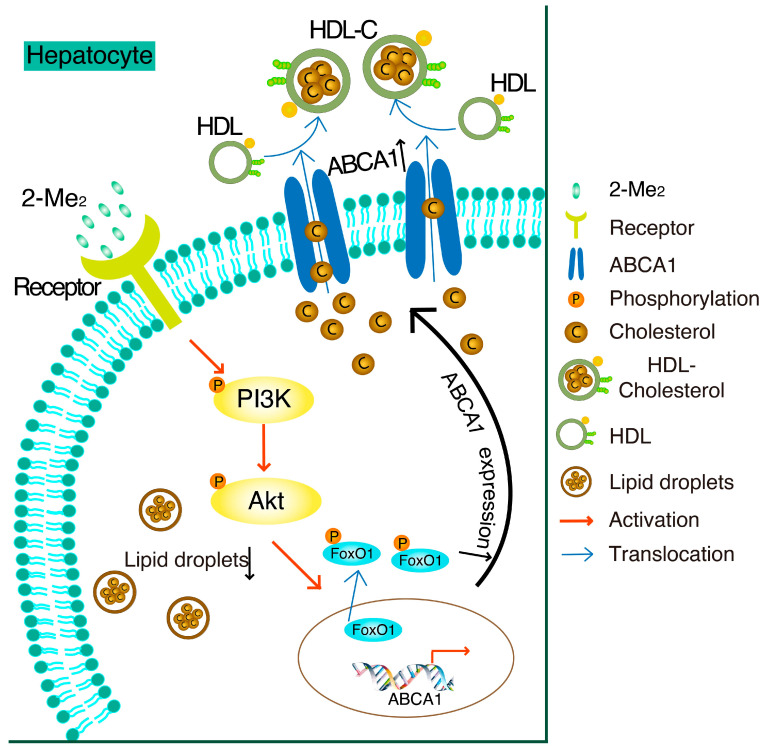
Schematic diagram of the mechanisms in 2-ME_2_-suppressed cholesterol accumulation by the upregulation of ABCA1 in HepG2 cells.

In this study, we found that 2-ME_2_ upregulated the expression of hepatic ABCA1 through the PI3K/Akt/FoxO1 signaling pathway (shown in Figure 6). ABCA1 is a membrane protein that plays an important role in HDL formation and in cholesterol efflux. Active transport via transporters, including the best characterized ABCA1, ABCG1, and SR-BI, is mainly responsible for the bulk efflux of cholesterol from the cells onto extracellular acceptors. Here, we reported that 2-ME_2_ induces the expression of ABCA1 and lowers the intracellular cholesterol content, but how the cholesterol efflux is involved in this mechanism is for further study. The dysfunction of ABCA1 in Tangier disease results in cholesterol accumulation in the peripheral tissue and causes severe HDL deficiency [5]. Typically, a specific hepatic ABCA1 knockout decreases the generation of heterogeneous-sized nascent HDL particles in vivo, leading to large TG-rich very low-density lipoprotein (VLDL) accumulation in the liver [22]. Thus, ABCA1 has a beneficial effect to protect the liver from cholesterol accumulation. This is also confirmed by our recent study that the upregulation of hepatic ABCA1 expression by insulin-like growth factor-1 (IGF-1) or glucagon-like peptide-1 (GLP-1) reduced hepatic cholesterol accumulation in mice [19,20]. In this study, hepatic ABCA1 expression was elevated by 2-ME_2_, which contributed to the reduction of the lipid content in hepatocytes. As reported, GPER or GPR30 is a high-affinity membrane receptor of 2-ME_2_ and shows its protective effect on the cardiovascular system [23]. The global knockout of GPER increased the total and low-density lipoprotein-cholesterol (LDL-C) levels in mice, while the activation of GPER by its small molecule agonist rescued this effect [24]. Moreover, the deficiency of GPER accelerates liver inflammation and fibrosis [25]. However, the specific receptor of 2-ME_2_ responsible for these activities remains unknown. Thus, further studying needs to be done to investigate the receptor of 2-ME_2_ and its role in regulating hepatic lipid metabolism.

In HepG2 cells, treatment with 2-ME_2_ induces ABCA1 expression by activation of the PI3K/Akt pathway and phosphorylation of FoxO1. As a result, cholesterol accumulation is suppressed by 2-ME_2_.

Physiologically, the range of the plasma 2-ME_2_ level in women is 10–55 pg/mL (0.03–0.18 nM). During pregnancy, the level of 2-ME_2_ increases to 1000 times [26]. A previous study suggested that 2-ME_2_ at a physiological concentration may regulate some biological processes, and its higher dose may induce pathophysiological processes in tissues [1]. However, the concentration of 2-ME_2_ varies a lot in different tissues, and it is even higher in certain tissues than in the plasma [27]. Thus, we treated HepG2 cells with varying concentrations of 2-ME_2_, starting from the lower physiological dose (0.01 μM), and analyzed the protein and mRNA expression, as well as the transcription activity of ABCA1. Our results demonstrated that even a lower physiological concentration of 2-ME_2_ (0.1 μM) could stimulate the expression and transcription of ABCA1, and 1 μM of this metabolite exhibited the strongest effect. This corroborates the findings of a previous study that 1 μM of 2-ME_2_ significantly reduced the angiotensin type 1 receptor in rat liver epithelial and aortic smooth muscle cells [27]. Since the bioavailability of 2-ME_2_ is only 1 to 2% [28], the dosage needs to be carefully determined before the administration of 2-ME_2_ in vivo.

2-ME_2_ as the main metabolite of estradiol is generated by a sequential process of 17β-estradiol (E2) via the enzyme cytochrome P450 isoform 1A1 (CYP1A1) to produce 2-hydroxyestradiol (2HE) and then a conjugation reaction catalyzed by the enzyme catechol-O-methyltransferase (COMT) [29]. In humans, the COMT single-nucleotide polymorphism rs4680 (COMT^158Val-Met^) reduces the enzymatic activity and stability in Met allele carriers. COMT^158Val-Met^ is associated with many diseases, including diabetes, obesity, and fatty liver [30,31]. Genetic variations in COMT with lower enzymatic activity could be a trigger for the onset of metabolic syndromes and liver damage. Although entacapone, the inhibitor of COMT, is commercially available for the treatment of Parkinson’s disease and a few patients exhibit the onset of clinical apparent liver damage [32], this may be due to the short duration of this medication. The long-acting COMT inhibitor tolcapone has been associated with severe liver injury [33,34]. Further studying is required to understand the role of 2-ME_2_ on liver functions clinically.

One of the aims of this study was to investigate the signal transduction pathways activated by 2-ME_2_ in hepatocytes. Our results showed that the phosphorylation of Akt was significantly activated by 2-ME_2_, which is consistent with a previous report that 2-ME_2_ stimulates the PI3K/Akt pathway to induce the release of nitric oxide [35]. Our previous study proved that the activation of the PI3K/Akt pathway increases the transcription activity of ABCA1 in hepatocytes and pancreatic beta cells [16,19]. In this study, the results showed that the activation of the PI3K/Akt pathway by overexpression of Akt or the p110 catalytic subunit of PI3K remarkably enhanced the ABCA1 promoter activity. However, the inhibition of the PI3K/Akt pathway by its inhibitor, LY294002, or domain-negative Akt canceled the 2-ME_2_-induced ABCA1 promoter activity, confirming that activation of the PI3K/Akt pathway is required for the stimulation of ABCA1 transcription. However, in order to conclude that the activation of PI3K affects the expression of ABCA1, it will be necessary to conduct experiments with stable activated cells of PI3K in the future. Further, owing to the reduction of ABCA1 after inhibition of the PI3K/Akt pathway, the cholesterol content was increased compared to the treatment of 2-ME_2_.

The transcription factor forkhead box O (FoxO) family functions downstream of the PI3K/Akt pathway and plays an important role in maintaining the lipid homeostasis and metabolism in the liver [36]. Previous studies demonstrated that activation of the PI3K pathway suppressed the expression of FoxO1 in regulating the cell proliferation of several cancer cells [37,38]. Particularly in hepatocytes, the overexpression of FoxO1 induces the accumulation of triglycerides [39], and targeting the FoxO1 expression in mouse livers may contribute to reversed metabolic syndrome [40]. In our study, 2-ME_2_ decreased the expression of FoxO1, which was consistent with the reduction of the triglyceride content and total cholesterol content in HepG2 cells. Further, the phosphorylation of FoxO isoforms by the PI3K/Akt pathway led to their nuclear exclusion and inhibited the transcription of target genes. Previously, we reported that FoxO1 was phosphorylated by the activation of the PI3K/Akt pathway to regulate the IGF-1-mediated ABCA1 expression and hepatic cholesterol accumulation in growth hormone-deficient mice [19]. In the present study, FoxO1 was consistently phosphorylated by 2-ME_2_ via the PI3K/Akt pathway to stimulate the expression of hepatic ABCA1. In addition, 2-ME_2_ reduced the binding of FoxO1 to the ABCA1 promoter region to enhance the transcription of the ABCA1 gene. Further, the mutation of FRS in the ABCA1 promoter region or silencing of FoxO1 blocked the effect of 2-ME_2_ on ABCA1 expression. In clinical settings, the expression, nuclear accumulation, and dephosphorylation at Ser256 of FoxO1 were reported in patients with NASH, consistent with the reduction of Akt activation [41]. In addition, FoxO1 expression was stimulated by hepatitis C virus (HCV) infection to suppress the activation of Akt, which resulted in HCV-induced insulin resistance [42]. These reports suggested that the inhibition of hepatic FoxO1 may have a protective effect in the liver. However, FoxO1 was reported to repress LXR-mediated gene transcription via LXR responsive elements (LXREs) in HepG2 cells [43], and LXR is a well-known transcription factor that positively regulates ABCA1 gene transcription [44,45]. This evidence may explain the slight upregulation of the ABCA1 protein (about 10% upregulation compared to the siControl; *p* = 0.51) induced by the silencing of FoxO1 in Figure 5G. Thus, the detailed mechanism of FoxO1-regulated hepatic ABCA1 expression needs to be further investigated.

## 5. Conclusions

In summary, our findings showed that 2-ME_2_ reduces the lipid accumulation in hepatocytes through the elevation of hepatic ABCA1, which might be mediated by the PI3K/Akt/FoxO1 signaling pathway. This study may contribute to the understanding of how 2-ME_2_ protects the liver functions.

## Data Availability

The data that support the findings of this study are available from the corresponding author, K.J., upon reasonable request.
